# Invariant NKT cells facilitate cytotoxic T-cell activation via direct recognition of CD1d on T cells

**DOI:** 10.1038/s12276-019-0329-9

**Published:** 2019-10-25

**Authors:** Yingyu Qin, Sejin Oh, Sojung Lim, Jung Hoon Shin, Min Sang Yoon, Se-Ho Park

**Affiliations:** 1 Department of Life Sciences, Korea University, 145 Anam-ro, Seongbuk-gu, Seoul, 02841 Republic of Korea; 2 ImmunoMax Co., Ltd., KURBF Building, Korea University, 145 Anam-ro, Seongbuk-gu, Seoul, 02841 Republic of Korea

**Keywords:** Lymphocyte activation, Cytotoxic T cells

## Abstract

Invariant natural killer T (iNKT) cells are a major subset of NKT cells that recognize foreign and endogenous lipid antigens presented by CD1d. Although iNKT cells are characteristically autoreactive to self-antigens, the role of iNKT cells in the regulation of cytotoxic T lymphocytes (CTL) has been elucidated using α-galactosylceramide (α-GalCer), a strong synthetic glycolipid that is presented by professional antigen presenting cells (APCs), such as dendritic cells. Despite the well-known effects of α-GalCer and dendritic cells on lipid antigen presentation, the physiological role of endogenous antigens presented by CTLs during crosstalk with iNKT cells has not yet been addressed. In this study, we found that antigen-primed CTLs with transient CD1d upregulation could present lipid self-antigens to activate the iNKT cell production of IFN-γ. CTL-mediated iNKT cell activation in turn enhanced IFN-γ production and the proliferation and cytotoxicity of CTLs. We also found that the direct interaction of iNKT cells and CTLs enhanced the antitumor immune responses of CTLs. This partially explains the functional role of iNKT cells in CTL-mediated antitumor immunity. Our findings suggest that in the absence of exogenous iNKT cell ligands, iNKT cells enhanced the CTL production of IFN-γ and CTL proliferation and cytotoxicity via direct interaction with CD1d expressed on T cells without interacting with APCs.

## Cancer: Direct interaction between immune cells promotes anti-tumor responses

Cancer-killing T cells engage in a form of molecular crosstalk with other specialized immune cells to enhance anti-tumor immune responses in mice. Se-Ho Park and colleagues from Korea University in Seoul, South Korea, studied a specialized population of immune cells known as invariant natural killer T (iNKT) cells, which serve as important mediators of tumor surveillance. They showed that iNKT cells directly interact with cancer-killing cytotoxic T cells through surface molecules and not through other immune cells. The resulting activation of iNKT cells leads to the production of a pro-inflammatory signaling molecule, which in turn enhances the proliferation and killing potential of the cytotoxic T cells, ultimately producing more potent tumor control in a mouse model of lymphoma. The findings could aid in the development of iNKT-based cancer immunotherapies.

## Introduction

Natural killer T (NKT) cells are a subset of TCR αβ cells that recognize self and foreign lipids and glycolipid antigens presented by the MHC class I-like molecule CD1d. Invariant NKT (iNKT) cells represent a major population of cells expressing a unique invariant TCRα chain (Vα14Jα18 in mice and Vα24Jα18 in humans) and a limited number of variable TCRβ chains^1^. iNKT cells are classified as innate-like lymphocytes that promptly secrete Th1 or Th2 cytokines when antigens bind with TCRs^2–4^. Activated iNKT cells can regulate the adaptive immune response via the recruitment, activation, or modulation of the responses of NK cells, DCs, B cells, and T cells^4–8^. Many reports have suggested that the activation of iNKT cells enhances the CTL-mediated elimination of cancer cells or various infections^9–12^. However, most of these studies were focused on the activation of iNKT cells following stimulation with foreign glycolipids, such as the marine sponge-derived glycolipid α-galactosylceramide (α-GalCer), a strong and prototypical synthetic glycolipid^9,10,13–15^. In these immune responses, activated iNKT cells potentiate dendritic cells (DCs) in a CD40-dependent manner, which in turn enhances the CTL response. Although this mechanism has been well studied, nonphysiological activation using powerful synthetic glycolipid antigens may somehow evoke the differential activation of iNKT cells. Hence, the physiological role of iNKT cells in the immune responses of CTLs remains to be elucidated.

iNKT cells are characteristically autoreactive to self-antigens^16^ and show characteristic phenotypical activation and memory responses before encountering foreign antigens^2,3,16^. CD1d is constitutively expressed by many types of cells^17^. In addition to antigen presenting cells (APCs), T cells also express CD1d^18^. Tumor-specific CTLs loaded with α-GalCer can recruit and activate iNKT cells to promote CTL-mediated antitumor immune responses^19^. This suggests that it is possible that mature CD8^+^ T cells present self-ligands via CD1d to stimulate iNKT cells. Our previous study showed that iNKT cells can promote secondary immune responses of CTLs against tumor cells in the absence of exogenous ligands of iNKT cells^20^. Nevertheless, it is currently unclear how these homeostatic iNKT cells can strengthen the antigen-specific CTL response. In the present study, we observed that iNKT cells enhanced the effector functions (IFN-γ production, proliferation, and cytotoxicity) of CTLs via the direct recognition of endogenous antigens presented by CD1d on CTLs without the involvement of APCs, such as DCs, or exogenous iNKT cell antigens. Our findings suggest that antigen-stimulated CTLs can activate iNKT cells and cytokines produced by CTL-activated iNKT cells to play a role in the iNKT cell-mediated strengthening of CTL effector function.

## Materials and methods

### Mice

C57BL/6(B6) wild type (WT) and ovalbumin (OVA)_257–264_-specific TCR (Vα2 and Vβ5)-expressing transgenic mice (OT-1) were purchased from The Jackson Laboratory. Jα18 knockout (Jα18^KO^) mice were gifts from Dr. M. Taniguchi (RIKEN, Yokohama, Japan). CD1d knockout mice (CD1d^KO^) and Vα14 transgenic mice were provided by Dr. Albert Bendelac (University of Chicago, Chicago, IL, USA). CD1d^KO^ mice were crossed with CD1d^+/+^ (CD1d^WT^) OT-1 mice to obtain CD1d^+/−^OT-1 mice. CD1d^+/−^OT-1 mice were further backcrossed with CD1d^KO^ mice to obtain CD1d^KO^ OT-1 mice. Ly5.1^+/−^ (Ly5.1^+^Ly5.2^+^) OT-1 or Ly5.1^+/+^OT-1 mice were obtained from OT-1 mice that were crossed with congenic Ly5.1^+/+^ B6 mice. Ly5.1^+/−^ (Ly5.1^+^Ly5.2^+^) CD1d^KO^ OT-1 mice were obtained using the same method. All mice exhibited a C57BL/6 background. They were raised in specific pathogen-free conditions at Korea University and used at 7–9 weeks of age. The experimental protocols adopted in this study were approved by the Institutional Animal Care and Use Committee of Korea University.

### Cell preparation and enrichment by MACS

Splenic DCs were obtained from splenocytes of CD1d^KO^ mice via magnetic-activated cell sorting (MACS) using anti-CD11c micro beads (Miltenyi Biotec). iNKT cells were purified from Vα14Tg mice using an NK1.1^+^ iNKT sorting kit (Miltenyi Biotec). CD8^+^ T cells were positively sorted from the spleens of OT-1 mice via MACS using anti-CD8α micro beads (Miltenyi Biotec) after the depletion of DCs with anti-CD11c magnetic beads (Miltenyi Biotec) according to the manufacturer’s instructions. The purity of the cells sorted by MACS was observed in the following order: DCs > 80%, iNKT cells > 90%, and CD8^+^ T cells > 98% (Supplementary Fig.1).

### CFSE labeling of CD8^+^ T cells

CD8^+^ T cells were prepared from the spleens of OT-1 mice by MACS. Purified CD8^+^ T cells were further resuspended in carboxyfluorescein diacetate succinimidyl ester (CFSE) labeling buffer and labeled with 2.5 μM CFSE via incubation at 37 °C for 10 min followed by extensive washing with RPMI 1640 medium.

### Reagents and in vitro stimulation

The cells were cultured in RPMI 1640 medium (Gibco) supplemented with 10% heat-inactivated fetal bovine serum (HyClone), 2 mM l-glutamine, 50 U/mL penicillin, 50 μg/mL streptomycin, 10 μg/mL gentamycin sulfate, and 50 μM β-mercaptoethanol. Chicken OVA (OVA_257–264_ [H-2Kb restricted; amino acid sequence, SIINFEKL) peptide was purchased from Genscript, and the OVA_257–264_/H-2K^b^ monomer (hereafter K^b^OVA) was obtained endogenously. An total of 5 μg/well (50 μl) of K^b^OVA was incubated at 4 °C overnight in 96-well-flat bottom plates (BD Biosciences).

CD1d^KO^ DCs (1 × 10^4^) were incubated with 10 μg/mL of OVA_257–264_ peptide for 18 h in a 96-well U bottom plate and washed with RPMI-1640 prior to the stimulation of the OT-1 CD8^+^ T cells. Purified OT-1 CD8^+^ T cells (1 × 10^5^) were stimulated with pretreated CD1d^KO^ DCs in the presence or absence of 5 × 10^4^ iNKT cells. Under DC-independent conditions, the purified OT-1 CD8^+^ T cells were stimulated with plates coated with K^b^OVA in the presence or absence of 5 × 10^4^ iNKT cells.

### In vitro cytotoxic assay

OT-1 CD8^+^ T cells were stimulated in plates coated with K^b^OVA for 18 h and cocultured with H^3^ thymidine-labeled E.G7 cells for an additional 8 h. OVA-expressing E.G7 cells (derivative of EL4) were provided by Dr. M. Mescher (University of Minnesota, Minneapolis, MN, USA). The specific lysis (%) was calculated using the following formula: specific lysis (%) = [(spontaneous CPM − experimental CPM)/spontaneous CPM] × 100.

### Flow cytometric (FACS) analysis

Cells were preincubated with anti-FcγR II/III monoclonal antibody (mAb, 2.4G2) and labeled for 30 min with the following mAbs (all mAbs are obtained from DB Biosciences if not specified otherwise): anti-CD8α-PerCP-Cyanine5.5 (clone 53-6.7), anti-CD1d-FITC (clone 1B1), anti-CD11c-FITC (clone HL-3), anti-TCR Vβ5.1-FITC or -APC (clone MR9–4), anti-CD69-FITC (clone H1.2F3), anti-Ly5.1-FITC (clone A20), anti-TCR Vα2-PE (clone B20.1), anti-CD3-PE (clone 17A2), anti-TCRβ-PE or PE-Cy7 (clone H57–597), anti-CD11b-PE (clone M1/70), anti-Ly5.2-PE (clone LG.7F9), CD44-PE (IM7), CD62L-APC(MEL-14), anti-IFNγ-APC (clone XMG1.2), and anti-IL-4-APC (clone 11B11). CD1d dimers were purchased from BD Biosciences, and α-galactosylceramide (α-GalCer) was purchased from Alexis. CD1d/α-GalCer dimers were prepared according to the manufacturer’s protocol.

Stained cells were analyzed on FACSCalibur and FACSVerse flow cytometers (BD Biosciences) using FlowJo software (FlowJo LLC). Figures with panel sets represent analyses performed in the same experiment to facilitate the direct comparison of the fluorescence intensity.

### Analysis of intracellular cytokine production

To determine the intracellular cytokine levels, cells were treated with Golgi Stop (BD Pharmingen) for the final 6 h of incubation. The cells were then initially stained for surface molecules with appropriate mAbs, fixed, and permeabilized using a Cytofix/Cytoperm kit (BD Pharmingen). They were finally stained with APC-conjugated anti-IL-4 or anti-IFN-γ mAbs for 45 min on ice. The percentage of cells expressing intracellular IL-4 or IFN-γ was determined via flow cytometry.

### OT-1 cell immune responses in vivo and tumor challenge models

Ly5.1^+/+^ CD1d^WT^ OT-1 CD8^+^ T cells (1 × 10^6^) and Ly5.1^+^Ly5.2^+^ CD1d^KO^ OT-1 CD8^+^ T cells (1 × 10^6^) were injected into WT (Ly5.2^+/+^) mice. One day after T-cell injection, the mice were immunized with OVA_257–264_ peptides/CFA or were not immunized. Two days after immunization, the mice were sacrificed and analyzed.

Sorted CD1d^WT^ OT-1 or CD1d^KO^ OT-1 CD8^+^ T cells (1 × 10^5^/mouse) were transferred into each recipient mouse via intravenous injection. One day after T cell transfer, the mice were challenged subcutaneously with 1 × 10^5^ E.G7 live tumor cells. Tumor growth was monitored every 3–4 days using digital calipers. The tumor mass was calculated using the following equation: tumor volume (mm^3^) = (length (cm) × width (cm)^2^) × 0.5.

### Statistical analysis

Statistical analysis was performed using GraphPad Prism 6.0 with either an unpaired *t*-test or one- or two-way ANOVA with Bonferroni’s multiple comparison test. The bars in all graphs represent the mean ± SEM. The significance is indicated with an asterisk: *p ≤ 0.05; **p ≤ 0.01; ***p ≤ 0.001; ****p ≤ 0.0001.

## Results

### iNKT cells promote CD8^+^ T-cell activation independent of DCs and exogenous NKT cell agonizts

**Fig. 1:**
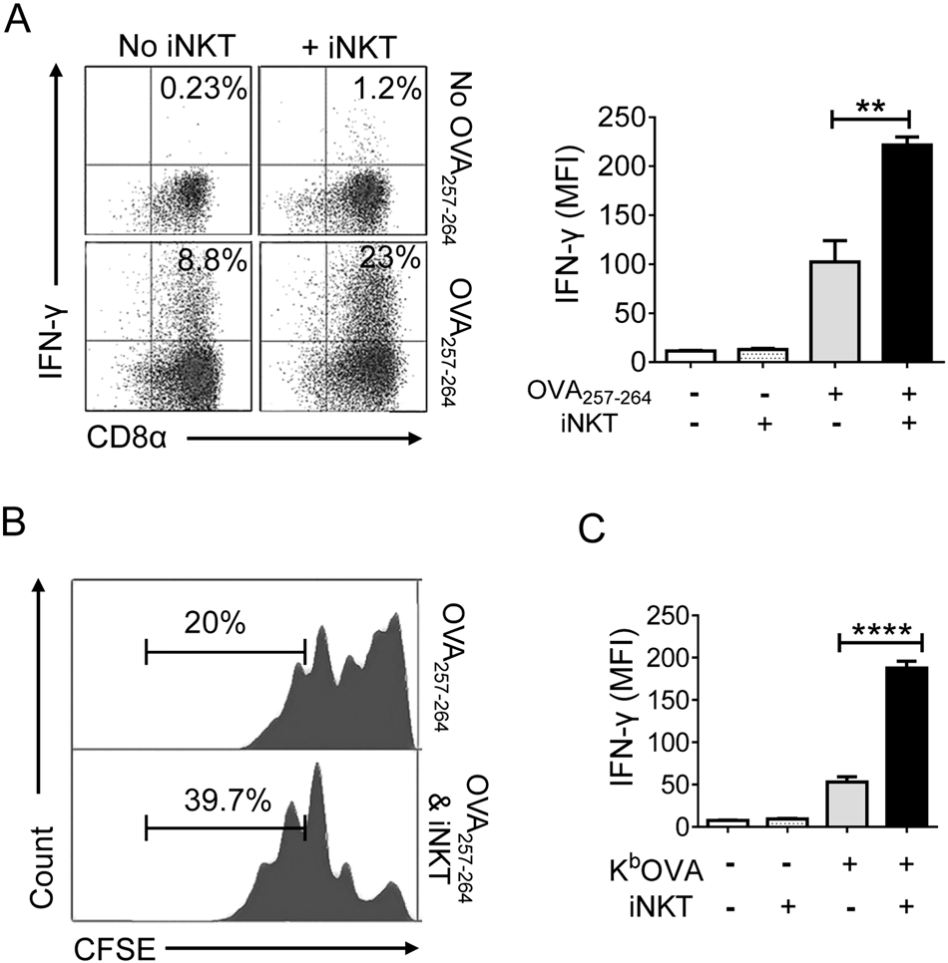
iNKT cells promote CD8^+^ T cell activation in the absence of exogenous NKT cell antigens. **a** Sorted OT-1 CD8^+^ T cells were activated by CD1d knockout DCs (CD1d^KO^ DCs) pretreated with OVA_257–264_ peptides in the presence or absence of iNKT cells. After two days of stimulation, the production of IFN-γ by CTLs was examined via intracellular FACS analysis. Detection of IFN-γ-producing CD8^+^ T cells by staining with anti-TCRVβ5.1, anti-CD8α, and anti-IFN-γ (left panel), **b** mean fluorescence intensity (MFI) of IFN-γ in OT-1 CD8^+^ T cells (right panel). **c** CFSE-labeled OT-1 CD8^+^ T cells were stimulated as described in (**a**). Representative histogram displaying CFSE dilution assay of OT-1 cells. **d** OT-1 CD8^+^ T cells were stimulated with plates coated with K^b^OVA in the presence or absence of iNKT cells. After 2 days of stimulation, the amount of IFN-γ produced by OT-1 cells was determined. All data shown are representative of four independent experiments with similar results. Data are presented as the mean ± SEM; **p < 0.01 and ****p < 0.0001. One-way ANOVA with Bonferroni’s multiple comparison test was used for data analysis

First, we examined whether iNKT cells directly promoted CD8^+^ T-cell activation or if CD8^+^ T cells were indirectly activated via interaction with DCs in the absence of exogenous iNKT cell ligands. OT-1 CD8^+^ T cells (purity > 98%) were stimulated with OVA_257–264_ peptide-pulsed DCs (purity > 80%) in the presence or absence of iNKT cells (purity > 90%, all purified cells shown in Supplementary Fig. 1). To exclude the possibility of iNKT cell activation by DCs, CD1d knockout DCs (CD1d^KO^ DCs) were used. After 2 days of stimulation, the OT-1 CD8^+^ T cells secreted higher levels of IFN-γ in the presence of NKT cells than the NKT cell-free culture group (Fig. 1a). Next, we examined whether iNKT cells also affected CD8^+^ T-cell proliferation using a CFSE dilution assay. The results showed that the proliferation of CTLs was increased when they were cocultured with iNKT cells (Fig. 1b). To exclude the effects of any costimulatory receptors or cytokines produced by CD1d^KO^ DCs on iNKT cells, we stimulated OT-1 CD8^+^ T cells with artificial APCs [from plates coated with OVA_257–264_ peptide/H-2K^b^ monomer (K^b^OVA)] with or without iNKT cells. The presence of iNKT cells also increased IFN-γ production by OT-1 cells even in the APC-free system (Fig. 1c). These results indicated that iNKT cells directly promoted CTL activation independently of DCs and exogenous iNKT cell ligands.

### Direct interaction of CTLs (CD1d) and NKT cells (TCR) promotes antigen-specific CD8^+^ T-cell activation

**Fig. 2:**
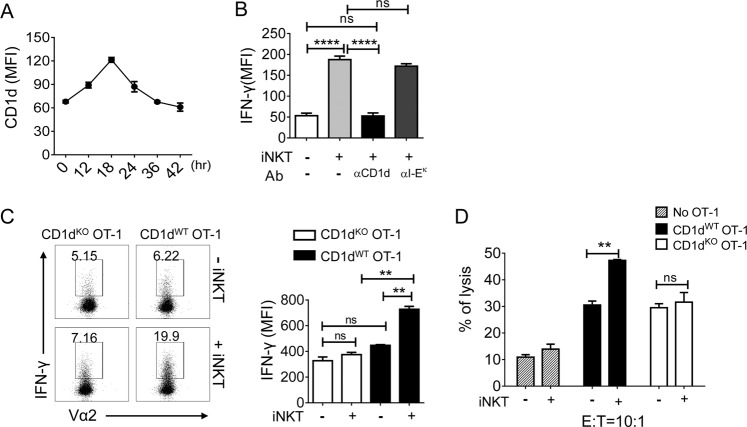
iNKT cells promote the effector function of CTLs via direct interaction with CD1d on CTLs. **a** OT-1 CD8^+^ T cells were stimulated with OVA_257–264_-loaded DCs, and the levels of CD1d were determined by FACS analysis at the indicated time points. **b** OT-1 CD8^+^ T cells were stimulated with plates coated with K^b^OVA in the presence or absence of iNKT cells supplemented with 25 µg/mL of anti-CD1d (20H2) or anti-I-E^k^ mAbs. After 2 days of stimulation, the levels of IFN-γ secreted by OT-1 cells were determined. **c** CD1d^WT^ OT-1 CD8^+^ T cells or CD1d^KO^ OT-1 CD8^+^ T cells were stimulated with K^b^OVA in the presence or absence of iNKT cells. After 2 days of stimulation, the levels of IFN-γ produced by OT-1 cells were determined by flow cytometry after intracellular staining. **d** OT-1 CD8^+^ T cells were stimulated with K^b^OVA in the presence or absence of iNKT cells for 18 h and cocultured with H^3^ thymidine-labeled E.G7 cells for an additional 8 h. The specific lysis (%) was calculated using the following formula: specific lysis (%) = [(spontaneous CPM − experimental CPM)/spontaneous CPM] × 100. All data are representative of at least three independent experiments. Data are presented as the mean ± SEM; **p < 0.01 and ****p *<* 0.0001. One-way ANOVA with Bonferroni’s multiple comparison test was used for data analysis

Since CD1d molecules are also expressed on T cells and upregulated upon PHA activation^18^, we analyzed the kinetic expression of CD1d on CD8^+^ T cells after TCR priming. The surface levels of CD1d peaked at 18 h after stimulation and declined thereafter (Fig. 2a). To test whether the effect of iNKT cells was mediated via the recognition of CD1d on OT-1 CD8^+^ T cells, OT-1 CD8^+^ T cells were pretreated with anti-CD1d mAbs (20H2, 25 μg/mL) before stimulation with K^b^OVA in the presence of iNKT cells. Pretreatment with anti-CD1d mAbs but not with anti-I-E^k^ mAb (nonspecific control) abrogated the effect of iNKT cells on the activation of CD8^+^ T cells (Fig. 2b). This result implied that, without APCs, CTLs were aided directly by iNKT cells via the interaction between CD1d on CTLs and TCRs on iNKT cells.

To rule out the possibility that DCs that had contaminated the purified iNKT cells stimulated iNKT cells and enhanced OT-1 activation, we compared the effects of iNKT cells on CD1d-expressing wild type OT-1 (CD1d^WT^ OT-1) and CD1d knockout OT-1 (CD1d^KO^ OT-1) CTLs. In the absence of iNKT cells, CD1d^KO^ OT-1 CTLs showed comparable reactivity to their cognate antigen OVA_257–264_ compared with that of CD1d^WT^ OT-1 CTLs (Fig. 2c). However, when iNKT cells were added to the cultures, the CD1d^WT^ OT-1 CTLs produced significantly higher levels of IFN-γ during K^b^OVA stimulation compared with the CD1d^KO^ OT-1 CTLs (Fig. 2c). These results indicate that iNKT cells promote IFN-γ production by CTLs via the direct interaction between CTL and iNKT cells, even in the absence of DCs. Next, we investigated whether this direct interaction affected the cytotoxicity of CTLs. The results showed that CD1d^WT^ OT-1 cells exhibited higher cytotoxicity following their interaction with iNKT cells. However, the cytotoxicity of CD1d^KO^ OT-1 cells was not affected by iNKT cells (Fig. 2d).

**Fig. 3:**
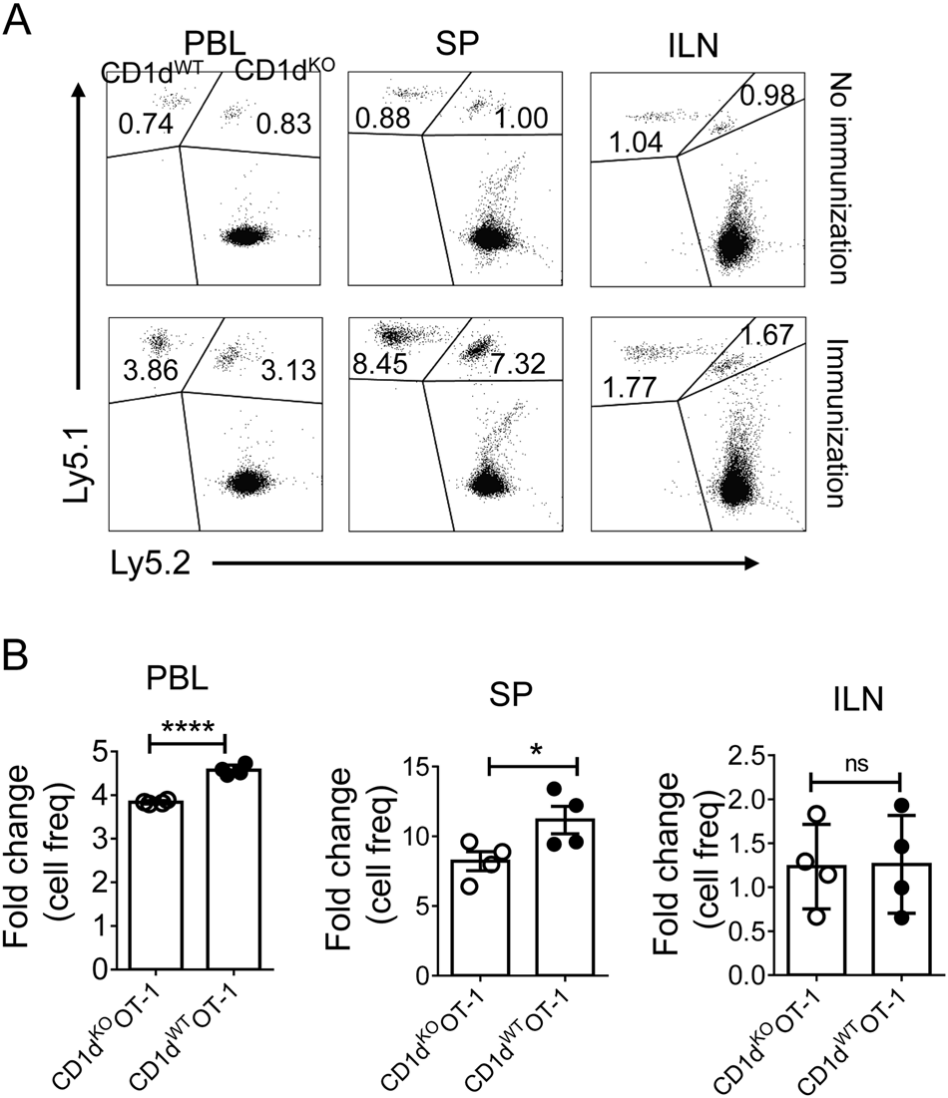
Direct interaction enhances the CTL response in vivo. Equal numbers of purified CD8^+^ T cells from Ly5.1^+/+^ CD1d^WT^ OT-1 splenocytes and Ly5.1^+^ Ly5.2^+^ CD1d^KO^ OT-1 splenocytes were i.v. cotransferred to Ly5.2^+/+^WT C57BL6/c mice (*n* = 6). After 1 day, 4 of 6 mice were stimulated with OVA_257–264_ peptides. After 2 days of stimulation, the transferred cells in peripheral blood (PBL), spleen (SP), and inguinal lymph nodes (ILN) were analyzed. **a** Representative FACS data show the frequencies of the donor cells. **b** The fold changes in donor cells were calculated by dividing the frequencies of donor cells in immunized mice by those in unimmunized mice. Each dot represents a recipient. Data are representative of two independent experiments. Data are presented as the mean ± SEM; *p < 0.05 and ***p *<* 0.0001. Unpaired *t* test was used for statistical analysis

To determine whether this direct interaction mediated CD8^+^ T cell immunity in vivo, the priming of CD8^+^ T cells was analyzed using congenic mice. We co-injected naïve CD1d^KO^ OT-1 cells (Ly5.1^+^Ly5.2^+^) and CD1d^WT^ OT-1 cells (Ly5.1^+/+^) into WT (Ly5.2^+/+^) mice and immunized the mice with OVA_257–264_ peptides. After 2 days of antigen-specific activation, the number of CD1d^WT^ OT-1 cells was slightly higher than that of CD1d^KO^ OT-1 cells in peripheral blood and spleen (Fig. 3a, b). There was no significant difference between the two populations in the inguinal lymph nodes (Fig. 3a, b), where the NKT cell population size was very small^21^. The difference in the cell proliferation of CD1d^WT^ OT-1 cells and CD1d^KO^ OT-1 cells was not as large as that observed under in vitro stimulation (Fig. 2b). This might be due to the lower abundance of iNKT cells in the in vivo environment compared to that under in vitro stimulation.

### Activated CTLs can activate iNKT cells, which in turn promote CTL activation

**Fig. 4:**
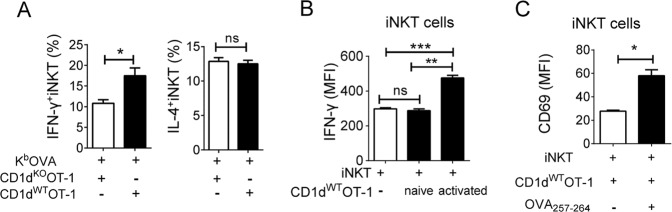
Direct interaction between iNKT cells and activated CD8^+^ T cells enhances IFN-γ production by iNKT cells. **a** Purified CD1d^WT^ OT-1 or CD1d^KO^ OT-1 CD8^+^ T cells were stimulated with K^b^OVA in the presence of iNKT cells. The percentages of IFN-γ- or IL-4-producing iNKT cells were examined using intracellular FACS analysis. **b** Naïve OT-1 CD8^+^ T cells were stimulated with K^b^OVA for 18 h. iNKT cells were cocultured with preactivated or naïve OT-1 cells. The levels of IFN-γ in iNKT cells were evaluated after 2 h of culture using intracellular FACS analysis (**b**). **c** Purified OT-1 CD8^+^ T cells and iNKT cells were cocultured with CD1d^KO^ DCs in the presence or absence of OVA_257–264_. After 18 h of activation, the surface expression of CD69 on iNKT cells was measured by FACS. iNKT cells were gated on the α-GalCer-CD1d dimer^+^ TCR-β^+^ cell population. All data are representative of at least three independent experiments. Data are presented as the mean ± SEM. *p < 0.05, **p < 0.01, and ***p < 0.001. Unpaired *t* tests (**a**, **c**). One-way ANOVA with Bonferroni’s multiple comparison test was used for statistical analysis (**b**)

iNKT cells are activated by endogenous lipid antigens presented by DCs in the presence of pro-inflammatory cytokines^22,23^, which prompted us to investigate the presentation of endogenous antigens by stimulated CTLs via CD1d to activate iNKT cells. First, we investigated whether OT-1 CD8^+^ T cells could activate iNKT cells via endogenous antigens presented on CD1d. iNKT cells were cocultured with CD1d^WT^ OT-1 or CD1d^KO^ OT-1 cells. The direct interaction between CD1d (CTLs) and TCR (iNKT) enhanced the iNKT cell production of IFN-γ but not of IL-4, another representative cytokine produced by iNKT cells (Fig. 4a). Next, we determined whether both naïve and primed CTLs activated iNKT cells. IFN-γ production and CD69 expression by iNKT cells were examined after coculture with naïve or activated OT-1 CD8^+^ T cells. We found that primed but not naïve CD8^+^ T cells activated iNKT cells (Fig. 4b, c). Taken together, these results suggest that primed CTLs present endogenous antigens via CD1d to activate iNKT cells to a certain degree.

**Fig. 5:**
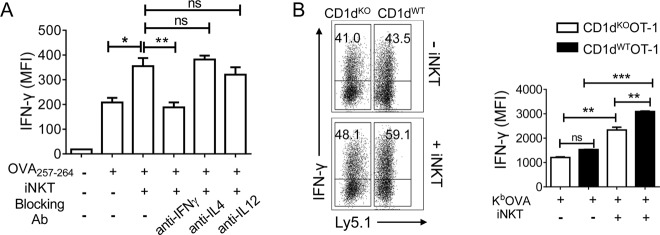
CTL-mediated activation of iNKT cells strengthens the effector function of CTLs. **a** OT-1 cells were cocultured with OVA_257–264_-unloaded or -loaded CD1d^KO^ DCs in the presence or absence of iNKT cells. After 12 h of stimulation, the indicated blocking monoclonal antibodies were added, and the cells were incubated for another 24 h. The levels of IFN-γ in OT-1 cells were examined using intracellular FACS analysis. **b** Equal numbers of Ly5.1^+^Ly5.2^+^CD1d^WT^ OT-1 and Ly5.2^+^Ly5.2^+^CD1d^KO^ OT-1 CD8^+^ T cells were mixed and stimulated with K^b^OVA in the presence or absence of iNKT cells. After 2 days of stimulation, the levels of IFN-γ in OT-1 cells were determined using intracellular FACS analysis. **b** MFI of the intracellular IFN-γ levels of CD1d^WT^ OT-1 or CD1d^KO^ OT-1 cells gated on Ly5.1^+^ (CD1d^WT^ OT-1) or Ly5.1^−^ (CD1d^KO^ OT-1) Vα2^+^CD8^+^ T cells. All data are representative of at least three independent experiments. Data are presented as the mean ± SEM. *p ≤ 0.05, **p ≤ 0.01, ***p ≤ 0.001. One-way ANOVA with Bonferroni’s multiple comparison test was used for statistical analysis

Next, we explored whether IFN-γ produced by iNKT was responsible for the iNKT cell-mediated enhancement of CTL effector function. The CTL response was observed by blocking cytokine expression. The abrogation of IFN-γ significantly reduced the iNKT cell-mediated increase in the CTL production of IFN-γ. However, blocking IL-4 or IL-12 did not affect the intracellular level of IFN-γ in CTLs (Fig. 5a). It appeared that IFN-γ played a role in the iNKT cell-mediated promotion of CTL function. However, CTLs themselves also synthesize IFN-γ. Blocking IFN-γ may also affect CTLs. Therefore, we activated populations of CD1d^KO^ (Ly5.2^+/+^) OT-1 and CD1d^WT^ (Ly5.1^+^Ly5.2^+^) OT-1 cells in the presence or absence of iNKT cells to determine whether cytokines released by CD1d^KO^ OT-1 cells activated iNKT cells to affect the CD1d^KO^ OT-1 cell production of IFN-γ. In the absence of iNKT cells, both CD1d^WT^ and CD1d^KO^ OT-1 cells showed similar IFN-γ production levels. However, the production of IFN-γ by CD1d^WT^ OT-1(Ly5.1^+^) cells was higher than that by CD1d^KO^ OT-1(Ly5.1^−^) cells in the presence of iNKT cells. In addition, the presence of iNKT cells increased the production of IFN-γ by CD1d^KO^ OT-1 but not as strongly as that by CD1d^WT^ OT-1 (Fig. 5b) because iNKT cells produced cytokines that influenced CD1d^KO^ OT-1 cells nonspecifically through a bystander effect, since both CD1d^WT^ OT-1 and CD1d^KO^ OT-1 cells were mixed in this experimental setting. These results suggested that the interaction between activated CTLs and iNKT cells depended specifically on the CD1d-TCR interaction. CTL-activated iNKT cells played a role in CTL function mediated by local soluble factors (mainly IFN-γ) via intimate physical contact with CTLs.

### Direct interaction promotes CTL-mediated antitumor effects

**Fig. 6:**
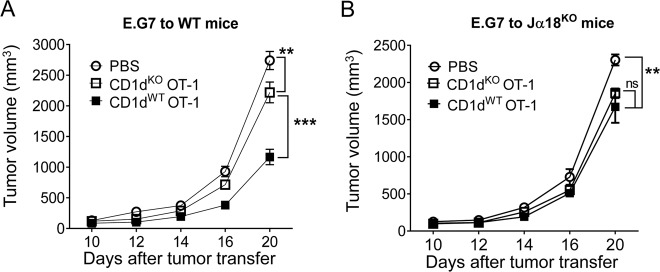
Direct interaction promotes CTL-mediated antitumor effects. CD1d^WT^ OT-1 CD8^+^ T cells or CD1d^KO^ OT-1 CD8^+^ T cells were intravenously transferred to wild type (**a**) and Jα18^KO^ NKT cell-deficient (**b**) mice (*n* = 5). Tumor growth was monitored every 2–4 days. The data shown are representative of two independent experiments with similar results. Data are presented as the mean ± SEM. **p < 0.01, ***p < 0.001; two-way ANOVA with Bonferroni’s multiple comparison test

Our previous study has shown that homeostatic iNKT cells can enhance CTL-mediated antitumor activity^20^. Here, we examined whether the direct interaction between CTLs and iNKT cells strengthened the CTL-mediated antitumor response. Sorted naïve CD1d^KO^ OT-1 cells or CD1d^WT^ OT-1 cells were transferred to wild type C57BL/6 mice, in which endogenous iNKT cells exist, followed by the injection of E.G7 tumor cells. CD1d^WT^ OT-1 cells efficiently controlled the growth of tumor cells. However, CD1d^KO^ OT-1 cells did not suppress tumor growth as efficiently as CD1d^WT^ OT-1 cells (Fig. 6a). To further establish that iNKT cells increased CD1d^WT^ OT-1 cell-mediated suppression of tumor growth, we performed the same experiments in Jα18^KO^ recipient mice, which lack iNKT cells. The results showed that the absence of iNKT cells eliminated the predominant tumor regression mediated by CD1d^WT^ OT-1 cells (Fig. 6b). Therefore, iNKT cells enhanced the antitumor effects of CTLs partially via the direct interaction of CTLs and iNKT cells.

## Discussion

NKT cells exhibiting both innate and adaptive immune functions play an antitumor role. They can also suppress infections^10–12^. The iNKT cell-mediated modulation of CTL activity is generally considered to occur via indirect regulation by DCs^9^. iNKT cells are activated by the CD1d-glycolipid complex presented by DCs and in turn induce the maturation of DCs to cross-prime CD8^+^ T cells. However, CD1d expression is not restricted to APCs. The expression of CD1d on CD8^+^ T cells is elevated after activation, indicating that CD1d expression on CD8^+^ T cells may play a role in the crosstalk between iNKT and CD8^+^ T cells. In the current study, we found that iNKT cells also promoted CD8^+^ T cell activation in a DC-independent manner, even in the absence of exogenous antigens (Fig. 1). Using CD1d^KO^ OT-1 cells, we found that iNKT cells directly interacted with CD1d on CTLs, which boosted IFN-γ synthesis by CTLs, cell proliferation, and cytotoxicity (Fig. 2). In addition, we found that the upregulation of CD1d expression on activated CD8^+^ T cells presenting endogenous antigens modestly activated iNKT cells to secrete IFN-γ, which might play a role in the enhancement of CTL function. Furthermore, we found that this direct interaction enhanced CTL proliferation and CTL-mediated antitumor immunity in in vivo CTL activation and established tumor models (Fig. 3 and Fig. 5). Therefore, the direct effect of iNKT cells on CD8^+^ T-cell immunity partially explained the physiological role of iNKT cells.

Several studies have shown that endogenous lipid antigens can activate peripheral iNKT cells^22,24–26^. The endogenous lipid antigens of iNKT cells have a diverse nature and are generally divided into glycosphingolipids and phospholipids^16,25,27^. Several candidate endogenous lipid antigens have been proposed, including isoglobotrihexosylceramide (iGb3)^16^, β-glucosylceramide(β-GlcCer)^24,28^, α-glycosylceramides^27^, the peroxisome-derived ether-bonded compound lysophosphatidylethanolamine (pLPE), and lysophosphatidic acid (eLPA)^29^. All of these lipid self-antigens that have been experimentally proposed to be involved in NKT cell activation are presented by APCs (DCs and macrophages). CTLs might also present these lipid candidates to activate iNKT cells. In fact, we found that CD1d^WT^ CTLs could activate iNKT cells, while CD1d^KO^ CTLs failed to activate iNKT cells, indicating that CTLs could activate iNKT cells by presenting endogenous antigens (Fig. 4).

In addition, the induction of CD1d may be important for augmenting NKT cell activation by weak endogenous antigens^30^. As shown in Fig. 2a, CTLs showed the transient elevation of CD1d expression after TCR engagement. Primed CTLs with a higher level of CD1d increased iNKT cell production of IFN-γ, indicating that the upregulation of CD1d might facilitate the presentation of endogenous antigens to iNKT cells via the additional synthesis of cytokines such as IL-2 and IFN-γ (Fig. 4a). The engagement of CD1d by CTLs enhanced the production of IFN-γ but not the production of IL-4 by iNKT cells (Fig. 4a). This modest effect on activation is consistent with the indirect activation of iNKT cells by DCs^23^.

iNKT cells play a significant role in the rapid production of cytokines to facilitate adaptive immunity^6,7^. Therefore, we investigated whether cytokines produced by CTL-activated iNKT cells enhanced CTL function. As shown in Fig. 5a, blocking IFN-γ but not IL-4 or IL-12 abolished the iNKT cell-induced increase in IFN-γ levels in CTLs. However, CTLs themselves also produced IFN-γ. Therefore, we generated a specific cytokine environment by using CD1d^WT^ OT-1 cell-activated iNKT cells and CD1d^KO^ OT-1 cells via coculturing these two types of OT-1 cells in the presence of iNKT cells. As shown in Fig. 5b, iNKT cells not only enhanced CD1d^WT^ OT-1 cell production of IFN-γ but also slightly enhanced production by CD1d^KO^ OT-1 cells, suggesting that the iNKT cell-mediated enhancement of CTL effector function was specifically induced by CD1d-dependent cell–cell contact. The slight increase in IFN-γ production by CD1d^KO^ OT-1 cells in the coculture system was attributed to the bystander effect of cytokines produced by iNKT cells. Figure 5a, b indicates that among the cytokines produced by iNKT cells, IFN-γ most likely enhanced CTL effector function, including the motility and cytotoxicity of CTLs^31^.

iNKT cells can interact with CD1d molecules^17^. This not only results in iNKT cell activation but also induces the phosphorylation of CD1d, intracellular signaling, and the release of cytokines from CD1d-bearing cells^32^. Hence, it is possible that the intracellular signaling of CD1d-expressing T cells is enhanced after interaction with iNKT cells.

**Fig. 7:**
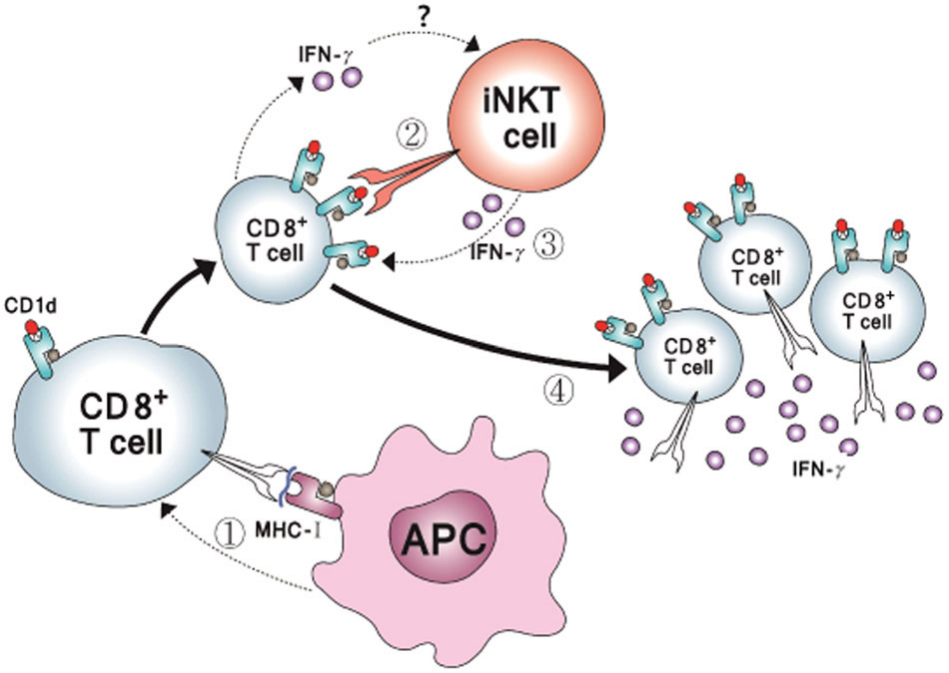
Model of the iNKT cell-driven optimization of CD8^+^ T cell activation. CD8^+^ T cells upregulate the expression of CD1d on their surfaces upon the recognition of cognate antigens (1). iNKT cells interact with CD1d on antigen-recognized CD8^+^ T cells to become activated (2) and to further stimulate CD8^+^ T cells in turn (3). CD8^+^ T cells reach the optimal level of activation and downregulate CD1d expression on the cell surface (4)

Taken together, our findings suggest that CD8^+^ T cells are activated via the recognition of specific antigens on APCs or target cells, leading to the upregulation of CD1d on activated CTLs. iNKT cells can directly interact with CD1d expressed on CTLs and boost the function of CTLs via the upregulation of IFN-γ production, cell proliferation, and cytotoxicity, suggesting that CD8^+^ T cells can reach maximal reactivity through a dual mechanism first involving the engagement of APCs followed by interaction with iNKT cells (Fig. 7).

## Supplementary information

Spplemental figure
