# Supplementary material for: Invariant NKT cells facilitate cytotoxic T-cell activation via direct recognition of CD1d on T cells
Source: Exp Mol Med. 2019 Oct 25;51(10):126. doi: 10.1038/s12276-019-0329-9 (PMC6814837; doi:10.1038/s12276-019-0329-9)
Supplement: Supplementary file 1 — Spplemental figure [file 12276_2019_329_MOESM1_ESM.docx]

Supporting information Fig.1


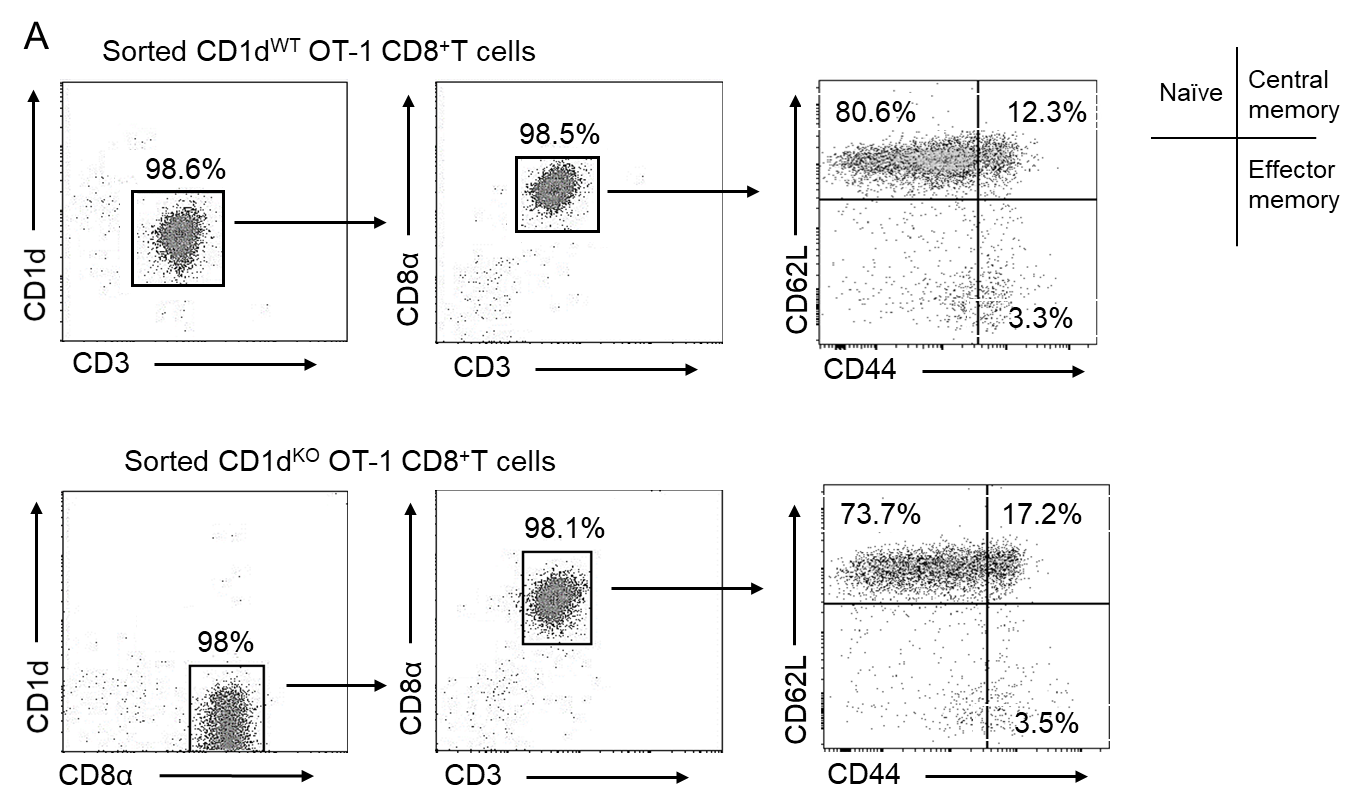


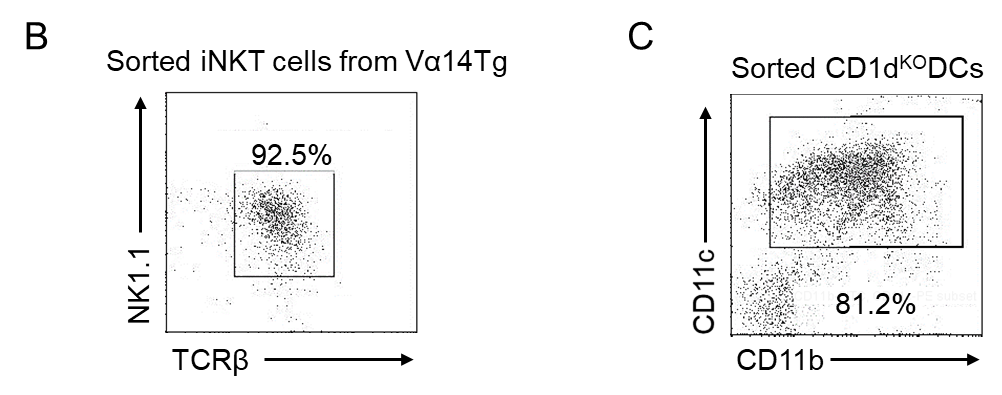


A, The purity and phenotype of sorted CD8^+^ T cells from CD1d^WT^ OT-1 mice (upper panel) or CD1d^KO^ OT-1 mice (lower panel).

B, The purity of sorted iNKT cells from Vα14 Tg mice.

C, The purity of sorted DCs from CD1d knock out mice.
